# *Elizabethkingia anophelis* Infection in Infants, Cambodia, 2012–2018

**DOI:** 10.3201/eid2602.190345

**Published:** 2020-02

**Authors:** Thomas A.N. Reed, Gabriella Watson, Chheng Kheng, Pisey Tan, Tamalee Roberts, Clare L. Ling, Thyl Miliya, Paul Turner

**Affiliations:** Angkor Hospital for Children, Siem Reap, Cambodia (T.A.N. Reed, G. Watson, C. Kheng, P. Tan, T. Miliya, P. Turner);; Lao-Oxford-Mahosot Hospital, Vientiane, Laos (T. Roberts);; Mahidol University, Mae Sot, Thailand (C.L. Ling); University of Oxford, Oxford, UK (C.L. Ling, P. Turner)

**Keywords:** *Elizabethkingia anophelis*, bacteria, neonatal sepsis, antimicrobial resistance, Cambodia, Thailand, Laos

## Abstract

We describe 6 clinical isolates of *Elizabethkingia anophelis* from a pediatric referral hospital in Cambodia, along with 1 isolate reported from Thailand. Improving diagnostic microbiological methods in resource-limited settings will increase the frequency of reporting for this pathogen. Consensus on therapeutic options is needed, especially for resource-limited settings.

*Elizabethkingia anophelis* is a recently identified aerobic, nonmotile, oxidase-positive, indole-positive species of gram-negative bacillus (*1*,*2*) that has been implicated in nosocomial and community outbreaks and associated with high mortality rates (*3*,*4*). We report a case of *E. anophelis* bacteremia in an infant in Cambodia in October 2018 and a retrospective study to identify previously misidentified isolates and describe the clinical features of *E. anophelis*–associated pediatric illness in Cambodia.

## The Study

A 7-day-old girl was brought to her local hospital with difficulty in breathing and poor feeding. She was a twin, born vaginally at 36 weeks’ gestation, with no antenatal or delivery complications. Both her mother and twin were in good health. Hospital staff administered ampicillin (50 mg/kg 2×/d) and gentamicin (5 mg/kg/36 h). 

The patient was transferred to the pediatric intensive care unit of Angkor Hospital for Children, a nongovernmental pediatric hospital in Siem Reap, Cambodia, with presumed late-onset neonatal sepsis. Upon arrival, she was cyanotic with recurrent apneas, requiring intubation, and had jaundice. Clinical examination and vital signs were otherwise unremarkable. Blood tests showed a leukocyte count of 8.5 × 10^9^/L (neutrophils 6.4 × 10^9^/L); hemoglobin 16.9 g/dL; platelet count 45 × 10^9^/L; C-reactive protein 195 mg/L; and total bilirubin 252 μmol/L. Lumbar puncture was omitted due to thrombocytopenia. A blood culture was transferred with her from the local hospital.

The day after transfer, she experienced symptoms of meningitis, including fever and seizures. We initiated anticonvulsant therapy and changed her antimicrobial therapy to intravenous meropenem (40 mg/kg 3×/d). Blood culture microscopy subsequently showed gram-negative bacilli, identified as *E. anophelis* on hospitalization day 3 by matrix-assisted laser desorption/ionization time-of-flight (MALDI-TOF) mass spectrometry using bioMérieux VITEK MS in an in vitro diagnostic mode using the spectrum knowledge base version 3.2.0 (bioMérieux, https://www.biomerieux.com). At this stage antimicrobial drugs were changed to intravenous ciprofloxacin (10 mg/kg, 2×/d) and vancomycin (15 mg/kg, 1×/d); a blood culture collected before the change confirmed bacteremia caused by *E. anophelis*.

The patient was extubated on day 6 and underwent lumbar puncture because her platelet count had improved. Cerebrospinal fluid was cloudy, with a leukocyte count of 265 cells/µL (75% polymorphs), glucose of 1 mmol/L, and protein of 13 g/L. Gram stain microscopy revealed no organisms, and culture was negative. After 28 days of ciprofloxacin/vancomycin, she was clinically well and discharged home.

At her 1-month follow-up appointment, she displayed clinical features of raised intracranial pressure, including neurologic deficits. Cranial ultrasound showed hydrocephalus, a suspected sequela of meningitis, and she was referred for neurosurgical opinion.

After the case described was identified, we retrieved all isolates in −80°C storage that had been identified since January 2012 as *Chryseobacterium meningosepticum*, *C. miricola*, or *Elizabethkingia* spp. We included in our study the first isolates from a given clinical episode: 4 identified as *C. meningosepticum*, 3 as *E. meningoseptica*, and the isolate already identified as *E. anophelis*. From subculture, we analyzed these using VITEK MS MALDI-TOF mass spectrometry. We identified 6 isolates as *E. anophelis* and 1 as *E. meningoseptica*. MALDI-TOF was unable to return an identification for 1 isolate, previously identified as *C. meningosepticum*.

We determined MICs to antimicrobial drugs for all *E. anophelis* isolates using Etest (bioMérieux). MIC_50_ result for ceftriaxone was 64 µg/mL; for sulfamethoxazole/trimethoprim, 0.25 µg/mL; for ciprofloxacin, 0.5 µg/mL; and for vancomycin, 12 µg/mL (Table).

**Table:** Characteristics of *Elizabethkingia anophelis* isolates from Cambodia and Thailand*

Characteristic	Isolate no.						
	1 (this study)	2	3	4	5	6	7
Patient characteristics							
Sex	F	M	F	M	F	M	F
Age at admission	6 d	8 mo	15 wk	0 d	51 d	0 d	0 d
Concurrent condition	Prematurity†	Duodenal atresia	Failure to thrive	Prematurity†	Ventricular septal defect	Prematurity†	Prematurity†
Country	Cambodia	Cambodia	Cambodia	Cambodia	Cambodia	Cambodia	Thailand
Clinical features							
Diagnosis	Meningitis	VAP	Meningitis	Sepsis	VAP	Sepsis	Sepsis
Treatment	CIP/VAN	MER	CAX	AMP/GM	CIP	IMP	AMP/GM
Outcome	Survived	Died	Unknown‡	Died	Died	Survived	Died
Length of admission, d	31	16	1	5	79	35	25
Specimen details							
Collection date	2018 Oct	2018 Jan	2015 Aug	2013 Aug	2012 Sep	2012 Mar	2017 Apr
Specimen type	Blood	Respiratory secretion	Blood	Blood	Respiratory secretion	Blood	Blood
Hospitalization day collected	1	16	1	5	64	21	22
Isolate details							
First ID	*E. anophelis*	*E. meningoseptica*		*C. meningosepticum*			*E. meningoseptica*
Initial ID method	MALDI-TOF	MALDI-TOF	API 20NE	API 20NE	API 20NE	API 20NE	API 20NE
MIC (µg/mL)							
VAN	8	16	16	16	8	16	8
SXT	0.25	0.25	0.25	0.5	0.25	0.5	NA§
CAX	32	64	>256	64	64	>256	>256
CIP	1	0.5	0.5	0.5	0.5	0.5	1

*AMP, ampicillin; CAX, ceftriaxone; CIP, ciprofloxacin; F, female; GM, gentamicin; ID, identification; IMP, imipenem; M, male; MER, meropenem; SXT, sulfamethoxazole/trimethoprim; VAN, vancomycin; VAP, ventilator-associated pneumonia. †It was not possible to retrieve gestational age for all patients. ‡Patient left hospital against medical advice.  §Sulfamethoxazole/trimethoprim MIC testing not available in the Thailand laboratory.

To provide regional context for these results, 2 microbiology laboratories in Mae Sot, Thailand, and Vientiane, Laos, also reanalyzed stored clinical isolates as we described. In Mae Sot, a single isolate of *E. meningoseptica* from a neonatal blood culture was reidentified as *E. anophelis*. In Vientiane, 9 isolates of *C. meningosepticum* were reidentified as *E. meningoseptica*, and the identity of 1 *E. meningoseptica* isolate remained the same. 

## Conclusions

Although reports of *E. anophelis* are rare, cases are reported from countries in southern Asia, including Singapore (*3*), Taiwan (*5*), and Hong Kong (*6*). Our findings are consistent with reports of *E. anophelis* infection from other countries demonstrating it to be an opportunistic organism affecting more vulnerable patient groups (*6*). The mortality rate associated with *E. anophelis* is high (50%), and isolation of *E. anophelis* from blood in two thirds of the children in this study demonstrates its importance as a human pathogen.

Previous reports of community- and hospital-acquired *E. anophelis* infection among infants have proposed a range of transmission routes, including vectorborne (*anopheles* mosquitoes) (*1*,*2*,*7*), waterborne (*8*), and vertical transmission (*9*). With no temporal clustering, and with most cases occurring among older infants, we suspect that unidentified environmental reservoirs are possible sources of these cases.

Previously, studies relied on 16S rRNA testing to identify *E. anophelis*, with biochemical phenotypic methods unable to distinguish between *Elizabethkingia* spp. (*10*). Although this method provides high discriminatory power, its use in diagnostic microbiology is limited to established laboratory settings. It also requires highly trained staff to interpret results which are rarely available within a clinically useful timeframe. Until late 2017, oxidase-positive gram-negative isolates were identified at the microbiology laboratory at Angkor Hospital for Children by biochemical phenotypic methods (API 20NE, bioMérieux); identification is now done by MALDI-TOF mass spectrometry. Misidentification of *Elizabethkingia* spp. using biochemical methods has been reported (*2*,*6*); however, updated MALDI-TOF databases provide reliable differentiation (*10*). As the resolution that MALDI-TOF mass spectrometry provides in pathogen identification expands, and its use becomes available in low- and middle-income countries, we expect to see higher reported incidence of *E. anophelis* infection. Conversely, it may become apparent that the burden of *E. meningoseptica* is not as high as previously thought, with retrospective studies already showing *E. anophelis* as the predominant species of its genus (*6*,*10*,*11*). In our study, this possibility was not found to be the case in Laos, suggesting possible regional variation.

*E. anophelis* demonstrates phenotypic and genotypic resistance to multiple antimicrobial drugs, and, without epidemiologically based interpretive cutoffs, selection of therapeutic options is challenging (*4**,**5*,*10*,*12*). High MICs to ceftriaxone are consistent with β-lactam resistance reported elsewhere, and carbapenem resistance should also be expected (*4*,*5*,*10*,*12*). Following Clinical and Laboratory Standards Institute guidelines (M100–29; 2019) (*13*) for “other non-*Enterobacteriaceae*,” these isolates were susceptible to ciprofloxacin and sulfamethoxazole/trimethoprim. This finding is not consistent with other regional data that show greater rates of resistance to these drugs (*5**,**10*). *E. anophelis* has been shown to be susceptible to piperacillin/tazobactam and to rifampin (*4*,*10*), which were not tested against in this study and are not currently available as therapeutic options in the study setting. It is unusual for gram-negative organisms to exhibit susceptibility to vancomycin, and interpretation of MICs to this drug should be approached with caution. Use of Etest in this study was a methodological limitation; the preferred method of broth microdilution was not available.

In summary, updates of mass spectrometry platforms have enabled identification of clinical *E. anophelis* isolates in Cambodia and Thailand. As diagnostic microbiology capacity expands in low- and middle-income countries, further reports of this organism are expected. Because of the associated high mortality rates for this pathogen, consensus on therapeutic options for infection caused by *E. anophelis* is needed, especially in resource-limited settings with restricted choices for antimicrobial drugs.
